# The mTOR kinase inhibitor rapamycin enhances the expression and release of pro-inflammatory cytokine interleukin 6 modulating the activation of human microglial cells

**DOI:** 10.17179/excli2019-1715

**Published:** 2019-09-06

**Authors:** Natalia Cappoli, Daniele Mezzogori, Elisabetta Tabolacci, Isabella Coletta, Pierluigi Navarra, Giovambattista Pani, Cinzia Dello Russo

**Affiliations:** 1 Institute of Pharmacology, Università Cattolica del S. Cuore, Roma; 2 Institute of Human Physiology, Università Cattolica del S. Cuore, Roma; 3 Fondazione Policlinico Universitario A. Gemelli IRCCS, Roma -Institute of Genomic Medicine, Università Cattolica del S. Cuore, Roma; 4 Angelini RR&D (Research, Regulatory & Development) - Angelini S.p.A.; 5 Pharmacology Unit, Fondazione Policlinico Universitario A. Gemelli IRCCS, Roma -Institute of Pharmacology, Università Cattolica del S. Cuore, Roma; 6 Fondazione Policlinico Universitario A. Gemelli IRCCS, Roma - Institute of General Pathology, Università Cattolica del S. Cuore, Roma

**Keywords:** HMC3 microglial cells, rapamycin, IL-6, ROS, pro-inflammatory cytokines

## Abstract

Emerging evidence suggests the potential use of rapamycin in treatment of several neurological disorders. The drug readily crosses the blood brain barrier and may exert direct immunomodulatory effects within the brain. Microglia are the main innate immune cells of the brain, thus critically involved in the initiation and development of inflammatory processes at this level. However, there are conflicting data from rodent studies about the pharmacological effects of rapamycin on microglial inflammatory responses. Considering that rodent microglia display relevant biochemical and pharmacological differences compared to human microglia, in the present study we studied the effects of rapamycin in an experimental model of human microglia, the human microglial clone 3 (HMC3) cell line. Rapamycin was tested in the nM range both under basal conditions and in cells activated with a pro-inflammatory cytokine cocktail, consisting in a mixture of interferon-γ and interleukin-1β (II). The drug significantly increased II stimulatory effect on interleukin-6 (IL-6) expression and release in the HMC3 cells, while reducing the production of free oxygen radicals (ROS) both under basal conditions and in cells activated with II. Consistently with its known molecular mechanism of action, rapamycin reduced the extent of activation of the so-called 'mechanistic' target of rapamycin complex 1 (mTORC1) kinase and the total amount of intracellular proteins. In contrast to rodent cells, rapamycin did not alter human microglial cell viability nor inhibited cell proliferation. Moreover, rapamycin did not exert any significant effect on the morphology of the HMC3 cells. All together these data suggest that the inhibition of mTORC1 in human microglia by rapamycin results in complex immunomodulatory effects, including a significant increase in the expression and release of the pro-inflammatory IL-6.

## Introduction

Rapamycin (RAPA) is a fermentation product derived from *Streptomyces hygroscopicus*, which was isolated from a soil sample collected on Easter Island (Rapa Nui, hence the name) in 1976. It was originally characterized as a macrolide antibiotic with antifungal properties, but clinically used for its immunosuppressive activity, as in the prevention of organ transplant rejection and in the treatment of certain autoimmune diseases (Kelly et al., 1997[23]). In addition to immunosuppression, the drug displayed other potential therapeutic properties, including antitumor activity, neuroprotective and neuroregenerative effects, lifespan extension and anti-atherosclerotic effects (Yoo et al., 2017[53]; Kaeberlein and Galvan, 2019[21]; Liu et al., 2019[30]). At the molecular level, RAPA acts through inhibition of the mTOR kinase, that is the ‘mechanistic’ target of rapamycin. The mTOR kinase is currently regarded as a crucial regulator of the immune system, controlling both metabolism and function in the innate and adaptive immune cells (Jones and Pearce, 2017[20]). In mammalian cells, mTOR exists in two distinct complexes, mTORC1 and mTORC2. These two complexes share the catalytic subunit of mTOR and some protein interactors, whereas differ in other components. For example, the protein raptor (the regulatory-associated protein of mTOR) is selectively associated with mTORC1 (Laplante and Sabatini, 2012[24]). RAPA is a lipophilic compound which readily crosses cell membranes and is accumulated within the cells. In the intracellular compartment, RAPA blocks mTORC1 activity in a trimolecular complex with the immunophilin FKBP12 (FK506-binding protein of 12 kDa; also known as PPIase, FKBP1A) and the mTOR kinase linked via interaction with the FRB (FKBP12-rapamycin binding) domain. This interaction disrupts the association with raptor, uncoupling mTORC1 from its substrates and blocking downstream signaling (Hartford and Ratain, 2007[16]; Yip et al., 2010[52]). Interestingly, RAPA significantly crosses the blood brain barrier (BBB), which implies direct effects within the central nervous system (CNS) (Pong and Zaleska, 2003[39]). Notably, recent data suggest the involvement of the mTOR kinase in the regulation of brain inflammatory responses (Wang et al., 2018[47]; Saliba et al., 2017[41]). Accordingly, RAPA has been proposed as treatment for a variety of neurological disorders characterized by chronic inflammation within the CNS (Crino, 2016[8]). 

Chronic inflammatory processes in the CNS are mainly initiated and sustained by activation of microglial cells, the sentinel immune cells of the brain (Bachiller et al., 2018[2]). Interestingly, a direct link between mTOR and microglial pro-inflammatory activation was first observed *in vivo* in tuberous sclerosis complex brain lesions (Boer et al., 2008[5]). Histological analysis of the pathological regions confirmed cell-specific activation of mTOR in cortical tubers together with activated microglial cells and disruption of BBB permeability (Boer et al., 2008[5]). Consistently, a downstream target of mTORC1, the phospho-S6 ribosomal protein (p-S6RP) was significantly increased in microglial cells 24 h after traumatic brain injury (Park et al., 2012[36]). It was also shown that the PI3K/AKT/mTOR signaling pathway together with the hypoxia inducible factor-1α (HIF-1α) mediated the up-regulation of the inducible nitric oxide (NO) synthase (NOS2) in response to hypoxia, both in primary rat microglial cultures and in the mouse BV-2 microglial cell line (Lu et al., 2006[32]). Consistently, we have shown that mTORC1 activation is increased in rat primary microglial cells in response to different inflammatory stimuli (*i.e.* the bacterial endotoxin lipopolysaccharide LPS, or a mixture of pro-inflammatory cytokines) (Dello Russo et al., 2009[12]) or by the exposure to glioma conditioned medium (Lisi et al., 2014[29]). However, the role of mTOR in the regulation of microglial inflammatory responses is still not completely understood. For example, in our experiments we observed both anti-inflammatory and pro-inflammatory effects in response to RAPA. Namely, the drug reduced NOS2 activity and expression in response to cytokines; increased NOS2 expression, leaving significantly unaffected the enzymatic activity, in LPS-treated microglia; and significantly increased NOS2 expression and activity in glioma activated-microglial cells (Dello Russo et al., 2009[12]; Lisi et al., 2014[29]). On the other hand, the mTOR inhibitor RAD001 tended to reduce the cytosolic level of cyclooxygenase 2 (COX2) in microglial cells activated by pro-inflammatory cytokines, whereas it displayed significantly stimulatory effects on COX2 when administered in resting microglia (Dello Russo et al., 2009[12]). In catalase-exposed BV2 microglial cells, mTOR inhibition reduced both COX2 and NOS2 protein levels without affecting the mRNA steady state levels (Jang et al., 2005[19]). This effect was due to reduced activity of the mTORC1 downstream target, p70S6 kinase (p70S6K), which is a critical regulator of protein translation. In addition, reduction of NOS2 and interleukin 6 (IL-6) mRNA levels together with increased autophagic processes were observed in response to 100 nM RAPA in LPS-stimulated BV2 microglial cells (Han et al., 2013[15]). However, the mRNA level of other inflammatory genes, including IL-12, IFNγ, IFNβ, and TNFα, was increased by RAPA in this experimental model (Han et al., 2013[15]). Similarly, RAPA was shown to enhance the expression of both COX2 and the microsomal prostaglandin (PG) E synthase-1 and the release of PGE2 and PGD2 in rat microglial cells activated by LPS and poly(I:C) (de Oliveira et al., 2012[9], 2016[10]). In our studies, we also found that mTOR inhibition significantly reduces rat microglial viability *in vitro* under basal conditions (Dello Russo et al., 2009[12]). Similarly, it has been shown, using both primary cells and the EOC2 microglial cell line, that endogenous protection of microglial cells against oxygen-glucose deprivation relies upon the activation of the PI3K/AKT/mTOR pathway (Chong et al., 2007[7]). In addition, erythropoietin and Wnt1 promote anti-apoptotic signals in microglia via activation of the mTOR pathway, thus increasing cytoprotection (Shang et al., 2011[44]). Conversely, RAPA protective effects have been documented in LPS-activated microglial cells and the drug (at 100-1000 nM) significantly reduced microglial cell death (Han et al., 2013[15]). Taken together these observations suggest the involvement of mTOR in maintaining microglial survival under different conditions (*i.e.* resting cells and cell exposed to hypoxia), whereas the inhibition of the mTOR pathway resulted in microglial protective effects during inflammatory activation. Both the effects of RAPA on microglial survival and inflammatory activation appear to be complex and are not completely elucidated. 

In this regard, different molecular mechanisms, including transcriptional, translational and post-translational processes, may be involved and the effects (pro- *vs* anti-inflammatory; pro- *vs* anti-survival) may vary depending on the initial inflammatory stimulus or the inflammatory pathway considered. In addition, rodent microglia display important biochemical and pharmacological differences compared to human microglia (Smith and Dragunow, 2014[45]). Therefore, aiming to expand our previous observations, in the present paper we tested the effect of RAPA in the human microglial cell line HMC3. This cell line was established through SV40-dependent immortalization of a human embryonic brain-derived primary microglia culture (Janabi et al., 1995[18]) and it has been recently distributed by the American Type Culture Collection (ATCC), under the catalog designation of HMC3 ATCC®CRL-3304 (Dello Russo et al., 2018[11]). Consistently with data obtained in rodent models of microglia, RAPA displayed complex modulatory effects on human microglial functions. The drug significantly increases cytokine (IL-1β/IFNγ, II) dependent IL-6 expression and release, while significantly reduced ROS production, both under basal conditions and in presence of II. The expression of other inflammatory genes (*i.e.* TNFα, IL-1β, COX2, IL-8, MCP-1, TGFβ and ARG-1) was not significantly affected by RAPA treatment (unpublished results). In addition, we observed a significant reduction of the overall amount of cellular proteins in response to RAPA, which is consistent with the well-known inhibitory effect of RAPA on protein translation. The drug did not reduce microglial cell viability, neither displayed cytotoxic effects on human microglial cells after 24 h. Pro-inflammatory activation of microglial cells paralleled to changes in microglial morphology, including a significant increase in the percentage of cells with an elongated shape. However, the total cell number did not vary in response to II. RAPA did not exert any significant effect on cell number and morphology. Consistently the drug did not modify human microglial cell proliferation in 24 h experiments. All together these data confirm the involvement of mTOR in the regulation of human microglial inflammatory responses. Increased expression and release of IL-6 suggest a prevailing pro-inflammatory action, which can be beneficial in the context of tumor pathology, but it may result detrimental in other neurological disorders. 

## Methods

### Materials

The human microglial cell line, HMC3 (ATCC®CRL-3304, Lot/batch number 70002138) and cell culture reagents [(Eagle's Minimum Essential Medium (EMEM, ATCC® 30-2003™), and Fetal Bovine Serum (FBS, ATCC® 30-2020™)] were from the American Type Culture Collection (ATCC®, in partnership with LGC Standards S.r.l., Sesto San Giovanni, MI, Italy). Antibiotics were from Biochrom AG (Berlin, Germany). The Hanks Balanced Salt Solution (HBSS) was from Gibco (Thermo Fisher Scientific, Waltham, MA, USA). The recombinant pro-inflammatory cytokines, namely the human tumor necrosis factor α (TNFα, cat. n. RTNFAI) and the human interleukin-1β (IL-1β, cat. n. RIL1BI) were from Invitrogen (Thermo Fisher Scientific) and are produced in yeast. The lyophilized TNFα was reconstituted in 1 mL and IL-1β in 500 μl of certified endotoxin free water (G-Biosciences, St. Louis, MO, USA). Both cytokines were then diluted in 10 % FBS medium at the concentration of 10 µg/ml and stored in working aliquots at -80°C. Multiple freeze-thaw cycles were avoided. The human interferon-γ (IFNγ, cat. n. SRP3058) was purchased from Sigma-Aldrich (Saint Louis, MO, USA) and is produced in *E. Coli*. The compound is certified as containing <0.1 EU/μg endotoxin and it is suitable for cell culture studies. It was reconstituted in certified entotoxin free water (G-Biosciences) to a concentration of 1 mg/ml, further diluted to 1 μg/ml in 10 % FBS containing medium and stored in working aliquots at -80 °C. The mTOR inhibitor, RAPA was purchased from Calbiochem (distributed by Sigma-Aldrich, cat. n. 553210). Rabbit polyclonal anti-phospho [at ser-2448] mTOR was purchased from Novus Biological (Littleton, CO, USA, cat. n. 29745). Antibodies aimed at the detection of total mTOR (cat. n. 2972S) and phospho-p70S6K [at Thr-389] (cat. n. 9205S) were from Cell Signaling Technology (Danvers, MA, USA). The β-actin (ACTB) mouse monoclonal antibody (cat. n. 612656) was from R&D Systems (Minneapolis, MN, USA). Cell culture plastic-wares were purchased from Corning® (Corning, NY, USA) for the initial handling and characterization of the cell line. Subsequently, they were replaced by Orange Scientific (Braine-l'Alleud, Belgium) cell culture plates. For immunofluorescent assays carried out directly in cell culture plates, cells were seeded in black flat-transparent bottom 96-well plates (Greiner Bio-One International, Germany).

### Cell cultures

The HMC3 cell line was maintained according to the distributor's instructions, with some modifications introduced in our laboratory in order to improve cell viability (Dello Russo et al., 2018[11]). Cells were cultured in EMEM supplemented with 10 % FBS, and antibiotics (100 IU/mL of penicillin and 100 μg/mL of streptomycin). Cells were plated at the density of 20,000 cells/cm^2^ in 75 cm^2^ flasks, and kept in culture by changing the medium the day after plating and every 24-48 h. Cells were passaged twice a week, when reached 90-95 % confluency. For functional experiments, cells were plated at 30,000 cells/cm^2^, let recover overnight and stimulated the day after in complete growth medium, *i.e.* EMEM containing 10 % FBS and antibiotics. In these culture conditions, the HMC3 cells retain their morphological characteristics and express typical microglial markers, as reported in detail in our recent article (Dello Russo et al., 2018[11]).

### Free oxygen species measurements

Reactive free radicals were measured using H2DCF-DA [2,7-dichlorodihydrofluorescein diacetate (Invitrogen)]. At the end of the experiment, the incubation medium was replaced by Hanks' Balanced Salt Solution containing 20 μM of H2DCF-DA (Li et al., 2009[27]). Cells were incubated at 37°C for 1 h. The fluorescence signal due to H2DCF-DA oxidation within the cells was quantified using a microplate fluorescence reader (Victor-X^TM^4 microplate reader, PerkinElmer Inc, Waltham, Ma, USA), using 485 nm as excitation and 535 nm as emission wavelength.

### IL-6 measurements

The levels of IL-6 in the incubation medium were determined by the DuoSet ELISA development system (R&D Systems) specific for the human IL-6 according to the manufacturer's instructions. Briefly, aliquots of 25 μl of incubation medium for Control groups and 10 μl for II treated samples were assayed. A standard curve was generated within the concentration range of 9.38 - 600 pg/ml. The optical density of each well was quantified using a microplate reader (Victor-X^TM^4, PerkinElmer Inc) set to 450 nm with a wavelength correction to 570 nm. Readings for each standard, control, and sample were subtracted of the optical density of the zero standard or blanks (10 or 25 μl of plain medium not exposed to cell cultures). A standard curve was created generating a four parameter logistic curve-fit. 

### Cell viability measurement

Microglial viability was assessed by reduction of the tetrazolium compound (3-(4,5-dimethylthiazol-2-yl)-5-(4-sulfophenyl)-2H-tetrazolium, inner salt; MTS) contained in the CellTiter AQueous One Solution Reagent (Promega, Madison, WI, USA). For this assay cells were seeded in 96-well plates. At the end of the experimental procedure, 20 μL of MTS reagent were added to the cells that were further incubated at 37 °C for 3 h. Living cells bio-reduce yellow MTS into a purple soluble formazan product with an absorbance peak at 492 nm that was read in a spectrophotometer plate reader (iMark, Biorad, Hercules, CA, USA).

### Cell proliferation assay

Cell proliferation was evaluated with the DELFIA® Cell proliferation kit (PerkinElmer Inc). This is a non-isotopic immunoassay in which 5-Bromo-2'-deoxyuridine (BrdU) incorporation during DNA synthesis in proliferating cells is assessed by measuring europium fluorescence emission in a time-resolved fluorometer (Victor-X^TM^4, PerkinElmer Inc). For this assay, cells were seeded in black 96 well plates (in 100 μl medium/well). After 24 h treatments, 10 µl of BrdU labeling Solution (1 µM) were added directly to the cells (to a final concentration of 100 nM). Cells were further incubated for 4 h. Detection of BrdU was then performed according to the manufacturer's instructions.

### mRNA analysis in real time PCR

The expression of inflammatory genes was analyzed at the mRNA level by reverse transcription followed by real-time (Q)-PCR analysis, performed according to our standard protocol (Dello Russo et al., 2009[12]). Briefly, total cytoplasmic RNA was extracted from cells using TRIZOL reagent (Invitrogen) and 1 µg aliquots were converted to cDNA using random hexamer primers and the ImProm-II Reverse Transcriptase kit (Promega). Q-PCRs were performed using the following cycling conditions: 35 cycles of denaturation at 95 °C for 20 s; annealing at 60 °C for 30 s; extension at 72 °C for 30 s; the Brilliant SYBR Green QPCR Master Mix 2X (Agilent). The primers used were the followings: IL-6 (NM_000600.4) F394 (CCTTCCAAAGATGGCTGAAA) and R524 (TGGCTTGTTCCTCACTACT), which yield to a 150 bp amplicon; ACTB (NM_001101.4) F427 (TGGGACGACATGGAGAAA) and R573 (GAAGGTCTCAAACATGATCTGG), which yield to a 147 bp amplicon. Q-PCR reactions were carried out in a 20 μl reaction volume in a MX3000P real time PCR machine (Stratagene). Relative mRNA concentrations were calculated from the take-off point of reactions (threshold cycle, Ct) using the comparative quantitation method (Dello Russo et al., 2009[12]). Ct values for ACTB expression served as a normalizing signal. ACTB was selected considering that it is a constitutively highly expressed gene in human microglia (Gosselin et al., 2017[14]). Q-PCR efficiency ranged between 96 and 106 %. At the end of Q-PCR, the products were separated by electrophoresis through 2 % agarose gels containing 0.1 μg/ml ethidium bromide to ensure the product was correct in size.

### Sequencing analysis

**Figure 1:**
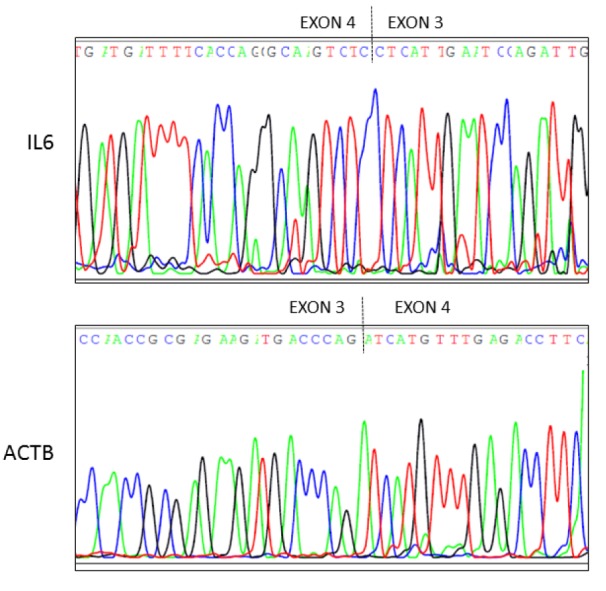
Sequencing electropherograms of IL-6 and ACTB cDNA derived by HMC3 microglial cells. Sequencing analysis of cDNA synthesized by HMC3 cells was performed using both forward and reverse primers for IL-6 and ACTB mRNA analysis. Top panel, it is shown the electropherogram of IL-6 (reverse primer). Bottom panel, it is shown the electropherogram of ACTB (forward primer). Primers used in real time (Q)-PCR to quantify IL-6 and ACTB transcripts spanned from exon 3 to exon 4 of the gene (dashed line).

cDNA was synthesized from HMC3 total cytoplasmic RNA, as already described. To verify the exact sequence of the IL-6 and ACTB Q-PCR products, cDNA was amplified through PCR using KAPA Taq ReadyMix PCR kit (KAPA Biosystems) and product were separated by electrophoresis through a 1.5 % agarose gel with ethidium bromide. The PCR products were purified by Illustra^TM^ ExoProStar^TM^ 1-Step (GE Healthcare, US77705V), according to the manufacturer's instructions. Purified amplification products were sequenced in both directions with BigDye Terminator v3.1 Cycle Sequencing kit (Life technologies, 4336917). Finally, sequencing products were purified with XTerminator^TM^ Solution Buffer (Life technologies, 4379323) before separation on a 3130 Genetic Analyzer (Life technologies). The primer sequences are those employed in Q-PCR. As shown in Figure 1(Fig. 1), sequence analysis of both PCR products confirmed that the amplified Q-PCR products corresponded to both human sequences (see Access numbers, Figure 1(Fig. 1)). 

### Western immunoblot

The HMC3 microglial cells were lysed in RIPA buffer (50 mM Tris-HCl, pH 8.0, with 150 mM sodium chloride, 1.0 % Igepal CA-630, also known as NP-40, 0.5 % sodium deoxycholate, and 0.1 % sodium dodecyl sulfate) (cat. n. R0278, Sigma-Aldrich) containing protease inhibitor cocktail diluted 1:100 (cat. n. P8340, Sigma-Aldrich,) and phosphatase inhibitor PhosStop, diluited 1:10 (cat. n. PHOSS-RO, Roche). The protein content in each sample was determined by Bradford's method (Biorad) using bovine serum albumin as standard. A 25-µg aliquot of protein was mixed 1:4 with 4X Laemmli Buffer (Biorad), boiled for 5 min, and separated through 7 % polyacrylamide SDS gels. Apparent molecular weights were estimated by comparison to colored molecular weight markers (Biorad). After electrophoresis, proteins were transferred to polyvinylidene difluoride membranes by semi-dry electrophoretic transfer (Biorad). The membranes were blocked with 5 or 10 % (w/v) low-fat milk, depending on the antibody used, in TBST (10 mM Tris, 150 mM NaCl, 0.1 % Tween-20, pH 7.6) (Biorad) for 1 h at room temperature. Subsequently, blots were incubated in the presence of the primary antibody overnight with gentle shaking at 4°C. Primary antibodies for phosphorylated mTOR (ser-2448), total mTOR, phosphorylated p70S6K (Thr-398) and ACTB were used at the final concentration of 1:1000. Primary antibodies were removed, membranes washed 3 times in TBST, and further incubated for 1 h at room temperature in the presence of specific secondary antibody, anti-rabbit IgG-HRP (Vector Laboratories, Burlingame, CA, USA) diluted 1:10,000 or anti-mouse IgG-HRP (Sigma-Aldrich) secondary antibody, diluted 1:8000. Following three washes in TBST, bands were visualized by incubation in ECL reagents (Pierce) and exposure to X-ray film (Thermo Scientific, USA).

### Cell morphology and counts

The morphology of HMC3 cells was analyzed by labeling of cytoskeletal F-actin filaments with rhodamine-phalloidin. Cells were plated at a density of 4,000 cells/cm^2^ on glass coverslips (12 mm diameter, 41001112 GmbH, Germany) in 24 well-plates (Orange Scientific) and let recover overnight. The following day, cells were treated for 24 h with medium alone or in presence of testing substances (as indicated in Figure 6A-D). At the end of the incubation period, cells were washed with phosphate buffered saline without Ca^2+^ and Mg^2+^ (PBS w/o, Biochrom) and fixed in 4 % (w/v) paraformaldehyde (Sigma-Aldrich) for 15 min. After fixation, cells were washed three times with PBS w/o, permeabilized with 0.25 % Triton-X-100 (Sigma-Aldrich) for 5 min and then washed for three times with PBS w/o. Rhodamine-phalloidin (cat. n. R415, Invitrogen), diluted 1:500 in PBS w/o, was added for 20 minutes. After incubation, DAPI (4',6-Diamidino-2-Phenylindole Dihydrochloride, cat. n. D1306, Invitrogen), diluted 1:2000 in PBS, was added to the cells for 10 min. Cells were then washed twice with PBS w/o. Coverslips were mounted using ProLong mounting media. Images of random fields were obtained in confocal microscopy (Olympus BX63) at 20x magnification. 10-15 images for each coverslip were acquired, and the merged images were used to count the cells for each condition. Total number of cells was counted using the nuclear DAPI staining by two different operators. All counted cells were classified according to their morphology (different shapes), as detected with the rhodamine-phalloidin staining. Different cell phenotypes are reported as percentage of controls (basal condition). Results are a representative of three different experiments.

### Data analysis

All experiments were done using 4-6 replicates per each experimental group and repeated at least 3 times. Data from three experiments were pooled for the analysis of ROS production, IL-6 release and cell viability and proliferation. Duplicates were used for the mRNA analysis, samples were assayed in triplicates, and the experiments were repeated at least twice. Western immunoblot experiments were carried out using a single replicate per each condition and were repeated at least two times with similar results. A representative blot is included in the paper. Band intensities were quantified using ImageJ software (National Institutes of Health) from autoradiographs obtained from the minimum exposure time that allows band detection. Background intensities were determined from an equal-sized area immediately above the band of interest and subtracted. Densitometry values for phosphorylated mTOR (Ser-2448) were normalized versus the total mTOR content, whereas values for phosphorylated p70S6K (Thr-398) were normalized using β-actin densitometry. Normalizing proteins were chosen based on their molecular weights, *i.e.* close to the phosphorylated protein of interest. Data are expressed as percentage of Control values that were set as 100 %. All data were analyzed by one- or two-way ANOVA followed by Bonferroni's post hoc tests; P values < 0.05 were considered significant.

## Results

### HMC3 inflammatory activation

**Figure 2:**
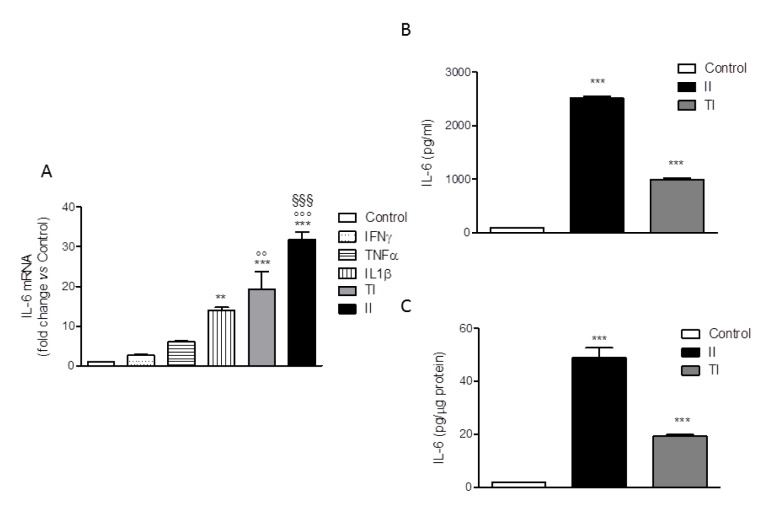
Effect of pro-inflammatory cytokines on IL-6 production by HMC3 cells. (A) HMC3 microglial cells were activated with different cytokines (IFNγ, TNFα, IL1β), each used at 10 ng/ml alone or in combination with IFNγ (IL-1β and IFNγ [II]; TNFα and IFNγ [TI]) for 24 h. Total cytosolic RNA was prepared from Control or activated HMC3 cells and used for real-time (Q)-PCR analysis of IL-6 gene expression. Data are expressed as fold change versus Control, taken as calibrator for comparative quantitation analysis of mRNA levels. Two replicates for each treatment group were measured in triplicate and the experiment was repeated three times with similar results. IL-1β significantly increased IL-6 gene expression, whereas IFNγ was not effective per se but significantly augmented the stimulatory effects of both IL-1β and TNFα. The combination with IL-1β (II) resulted as the most effective activator. Data are means ± SEM (n=6) and were analyzed by one-way ANOVA followed by the Bonferroni's *post hoc* test. ***, *P*<0.001, **P<0.01, versus Control; °°°*P*< 0.001, °°*P*< 0.01 versus TNFα alone; §§§ *P*< 0.001 versus IL-1β alone. (B-C) HMC3 microglial cells were stimulated with II and TI for 24 h and IL-6 levels in the incubation media were measured by a specific ELISA. At the end of the experiments, cells were lysed in 200 mM NaOH and protein content was evaluated by the Bradford's method. Data are shown as pg/ml (B) or were normalized by the total protein content of each well (pg/µg protein) (C). Results are from three independent experiments performed in triplicate. Both stimuli significantly increased IL-6 release. Data are means ± SEM (n=9) and were analyzed by one-way ANOVA followed by the Bonferroni's *post hoc* test. ***, *P*<0.001, versus Control

The human microglial HMC3 cell line was developed in the laboratory of Prof. Tardieu by immortalization of primary cultures of microglial cells derived from 6-8 weeks old human embryos (Janabi et al., 1995[18]). The cells were originally characterized for the ability to produce IL-6 under basal conditions and in response to the bacterial endotoxin, LPS or to the pro-inflammatory cytokine, IL-1. In addition, cells have been found to respond to IFNγ with the upregulation of several activation markers (for a recent review, Dello Russo et al., 2018[11]). Therefore, in order to select an optimal pro-inflammatory stimulus, we treated the HMC3 cells with different pro-inflammatory cytokines, including IFNγ, IL-1β and TNFα each used at 10 ng/ml alone or in combination for 24 h. The IL-6 mRNA intracellular level was taken as readout of inflammatory activation. As shown in Figure 2A(Fig. 2), IFNγ did not exert any significant effect per se whereas the other cytokines slightly and significantly (for IL-1β) increased the expression of IL-6. However, when used in combination with either IL-1β (II) or TNFα (TI), IFNγ significantly increased the stimulatory effect of these cytokines. In particular, a 30 fold increase in the intracellular levels of IL-6 mRNA was observed in response to II, whereas a 20 fold increase was detected in response to TI (Figure 2A(Fig. 2)). In addition, both II and TI significantly increased the secretion of IL-6 in the incubation medium after 24 h incubations (Figure 2B, C(Fig. 2)). Data are expressed as pg/ml or were normalized by the total protein content measured per each well (approximately 8 µg/10,000 cells plated in each well). None of the pro-inflammatory treatments modified the total protein content measured in each well in comparison to Control. Consistently, with the mRNA analysis the effect of II on IL-6 secretion (Figure 2B, C(Fig. 2)) was more pronounced than the stimulation obtained with TI. In addition, II significantly increased the expression of other inflammatory genes, including IL-1β, TNFα, COX2 (Dello Russo et al., 2018[11]), MCP1 and IL-8 (data not shown). The stimulatory effects of TI on the secretion of IL-6 after 24 h treatments were consistently lower in comparison to II (Figure 2B, C(Fig. 2)).

**Figure 3:**
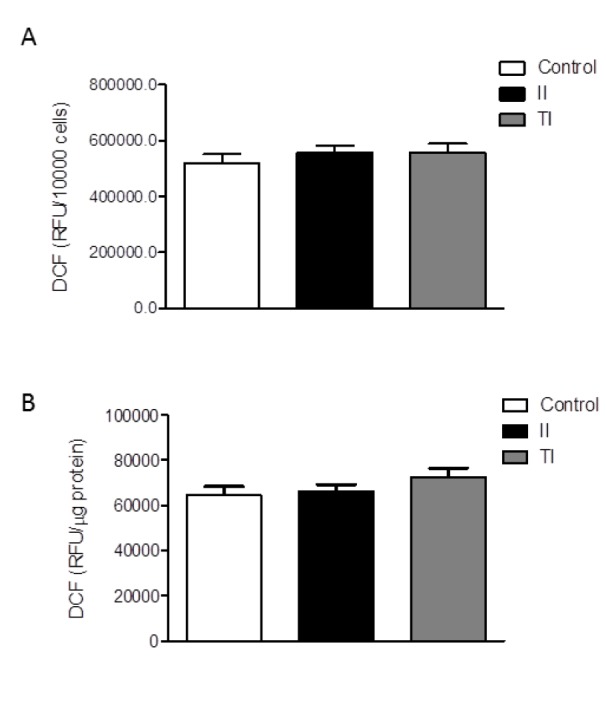
Effect of pro-inflammatory cytokines on ROS production by HMC3 cells. (A-B) HMC3 microglial cells were stimulated with II or TI for 24 h. The production of reactive free radicals (ROS) was assayed by the oxidation of H2DCF-DA (DCF) added to the cells at the end of the experiment. Cells were further incubated at 37 °C for 1 h, after which the fluorescence signal from each well was quantitated. Cells were subsequently lysed in 200 mM NaOH and protein content was measured by the Bradford's method. Data are shown as relative fluorescence units (RFU)/total cell number per well (i.e. RFU/10,000 cells) (A) or were normalized by total protein content per well (i.e. RFU/µg protein) (B). Results are pooled analysis of three independent experiments performed in triplicate. None of the treatments significantly affected basal ROS production in human microglial cells. Data are means ± SEM (n=9) and were analyzed by one-way ANOVA followed by the Bonferroni's *post hoc* test.

The HMC3 cells were also characterized for the spontaneous release of sizable amount of free oxygen radicals (ROS) (Li et al., 2009[27]; Jadhav et al., 2014[17]). The production of ROS by the HMC3 microglial cells was not significantly modulated by short term (2h) treatments with IFNγ (Li et al., 2009[27]), whereas a modest (0.15 fold) increase was induced by treatment with the TAT-C protein of the human immunodeficiency virus (HIV) (Jadhav et al., 2014[17]). Consistently, we observed a basal production of ROS in our cultures after 24 h incubation in plain medium (Figure 3A, B(Fig. 3)). Pro-inflammatory cytokines per se, at 10 ng/ml, did not modify basal levels of ROS production (data not shown), whereas both cytokine mixtures, II and TI, promoted a modest (0.1-0.2-fold) increase. However, this effect was not consistent among different experiments, carried out between passage 6 and 13 (Dello Russo et al., 2018[11]). A pooled analysis of three different experiments from two different thaws is shown in Figure 3(Fig. 3). Data are expressed as relative fluorescence units (RFU) per 10,000 cells (Figure 3A(Fig. 3)) or were normalized for the total protein content of each well (Figure 3B(Fig. 3)). When pooled together these results, we did not detect any significant increase in the production of ROS by HMC3 cells exposed for 24 h to either II or TI (Figure 3(Fig. 3)). 

### Rapamycin effects on HMC3 microglial pro-inflammatory activation

**Figure 4:**
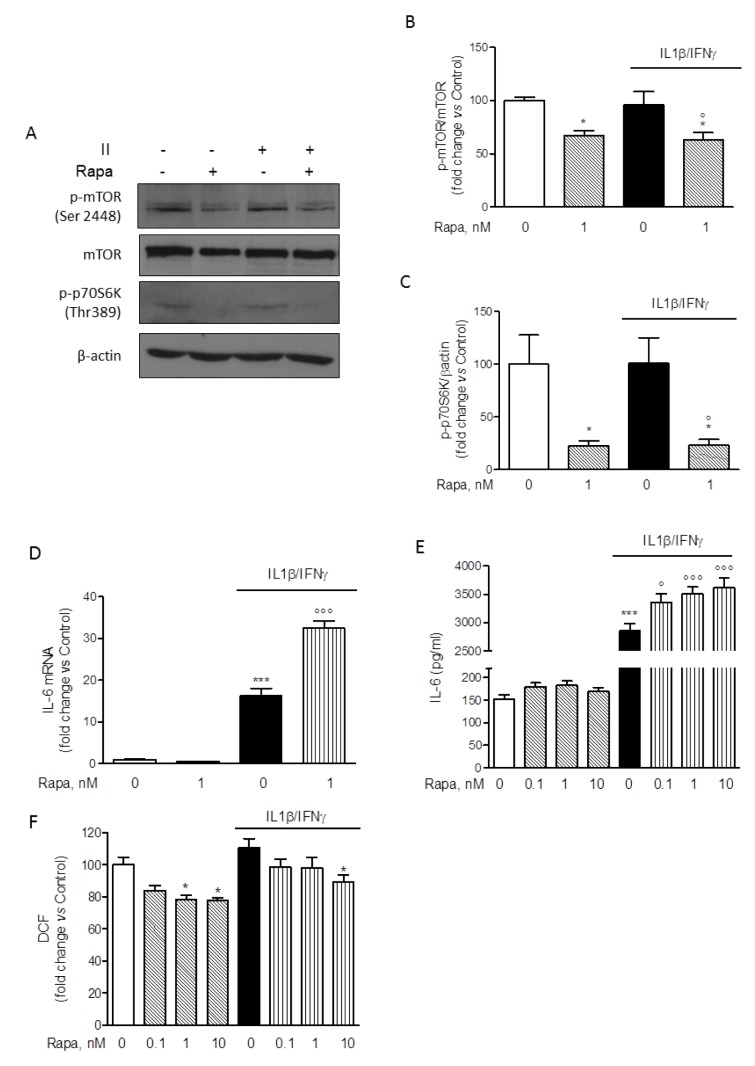
Modulatory effect of rapamycin on HMC-3 activation. (A) Whole-cell lysates were prepared from resting HMC3 cells or cells activated with II for 2 h. 1 nM rapamycin (RAPA) was added at the beginning of the experiment and equal amounts of proteins were analyzed by Western blot for phosphorylated mTOR kinase (p-mTOR Ser2448) and p-p70S6K (Thr389). Total mTOR and β-actin content was used as normalizer for each phosphorylated protein based on molecular weight. Blots are representative of three different experiments with similar results. (B-C) Quantification of phosphorylated proteins, p-mTOR and p-p70S6K, analyzed by Western blot. Data were normalized for total mTOR and β-actin content, respectively. Results are shown as percentage of control values, set as 100 %. Results are pooled analysis of at least two independent experiments and were analyzed by one-way ANOVA followed by the Bonferroni's post hoc test. *, *P*<0.05, versus Control; °, *P*<0.05, versus II. (D) HMC3 microglial cells were incubated in plain medium (as control) or activated with II for 24 h. 1 nM RAPA was added at the beginning of the experiment. Total cytosolic RNA was prepared and used for Q-PCR analysis of IL-6 gene. II significantly increased IL-6 expression in microglial cells. This stimulatory effect was further and significantly augmented by co-incubations with RAPA. The drug did not significantly modify basal IL-6 mRNA levels. Data are expressed as fold change versus Control, taken as calibrator for comparative quantitation analysis of mRNA levels. Each sample was measured in triplicate, the experiment was repeated three times with similar results. Data are means ± SEM (n=3) and were analyzed by one-way ANOVA followed by the Bonferroni's *post hoc* test. ***, *P*<0.001, versus Control; °°°, *P*<0.001, versus II. (E) Microglial cells were treated in plain medium (as Control) or stimulated with II for 24 h. RAPA in the concentration range of 0.1-10 nM was added at the beginning of the experiment. The amount of IL-6 released in the incubation media by HMC3 cells was measured. Consistently with mRNA data, II significantly increased the release of IL-6 in the incubation media and the stimulatory effect was further amplified by RAPA. Significant stimulatory action was observed at any level on concentration tested. Data are shown as pg/ml. Results are pooled analysis of three independent experiments (n= 4-6 per experimental group). Data are means ± SEM (n=15) and were analyzed by one-way ANOVA followed by the Bonferroni's *post hoc* test. ***, *P*<0.001, versus control; °, *P<*0.05, and °°°, *P*<0.001 versus II. (F) HMC3 cells were treated as reported above (E). The production of ROS was assessed by the oxidation of H2DCF-DA (DCF) added to the cells at the end of the experiment. II did not significantly affect ROS production in microglial cells, whereas RAPA significantly reduced ROS generation both in resting cells (at 1-10 nM) and in cells activated with II (at 10 nM). Data are shown as relative fluorescence units (RFU)/total cell number per well (i.e. RFU/10,000 cells). Results are pooled analysis of three independent experiments (n= 4-6 per experimental group). Data are means ± SEM (n=15) and were analyzed by one-way ANOVA followed by the Bonferroni's *post hoc* test. *, *P*<0.05, versus Control

Considering that II was the most effective activator of HMC3 microglial cells with respect to IL-6 expression and secretion (Figure 2(Fig. 2)), this combination was used in the subsequent experiments to test the effect of RAPA. Cells were initially exposed to II for different times (30, 60, 120, 240 min) and mTORC1 activation was evaluated by measuring the phosphorylation at Ser-2448 by Western immunoblot analysis. In contrast to primary rat microglial cultures, mTORC1 was activated under basal conditions in the HMC3 cell line, and no further activation was measured in response to II (data not shown, and Figure 4A(Fig. 4)). However, 1 nM RAPA significantly reduced the amount of mTORC1 activation both under basal conditions and in cells treated with II for 2 h (Figure 4B(Fig. 4)). Consistently, we detected in HMC3 microglial cells a basal activation of the mTORC1 downstream target p70S6K, as revealed by immunodetection of the protein phosphorylated at Thr-389, the specific site of phosphorylation by mTORC1 (Pearson et al., 1995[38]). Similar to mTORC1, activation of p70S6K was significantly reduced by 1 nM RAPA both under basal conditions and in cell treated with II for 2 h (Figure 4C(Fig. 4)). Taken together these data thus confirm the inhibitory activity of RAPA on the mTORC1 signaling pathway. Importantly, in these experimental conditions, 1 nM RAPA strongly enhanced the stimulatory effect of II on the expression levels of IL-6 mRNA (Figure 4D(Fig. 4)). Consistently, the drug was able to significantly increase the release of IL-6 stimulated by II, in the concentration range of 0.1-10 nM (Figure 4E(Fig. 4)). However, we did not observe any significant change in the expression levels of other inflammatory genes induced by II, including TNFα, IL-1β, COX2, MCP1, IL-8 (data not shown). The anti-inflammatory genes, TGFβ and ARG-1, were neither modulated by II (Dello Russo et al., 2018[11]) nor their expression changed in response to II and 1 nM RAPA (data not shown). In addition, II induced a slight reduction albeit not significant of BDNF expression that was reversed by RAPA (data not shown). On the other hand, RAPA significantly reduced ROS production in HMC3 cells both under basal conditions and in cells activated with II (Figure 4F(Fig. 4)). In contrast to RAPA, a known anti-inflammatory drug, dexamethasone (Dex), within the concentration range of 0.1-10 µM, significantly reduced the expression and the release of IL-6 (Supplementary Figure 1A-B) without affecting ROS production (Supplementary Figure 1C).

### Rapamycin effects on HMC3 protein content and cell viability

**Figure 5:**
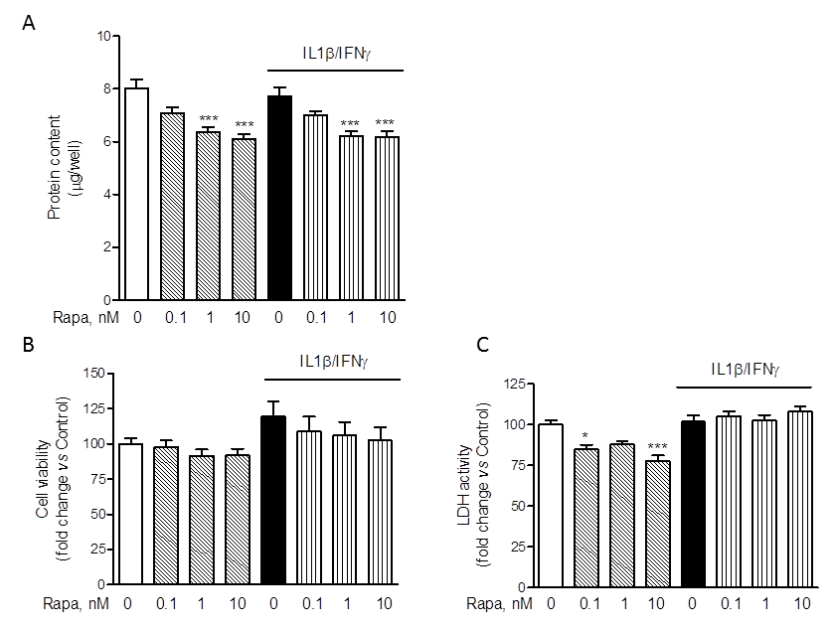
Effect of rapamycin on HMC-3 cell viability and protein synthesis. Microglial cells were stimulated with II for 24 h. RAPA in the concentration range of 0.1-10 nM was added at the beginning of the experiment. (A) At the end of experiments in which ROS production was assessed (Figure 4F), cells were lysed in 200 mM NaOH and protein content was evaluated by the Bradford's method. Results are pooled analysis of three independent experiments (n= 4-6 per experimental group). 1-10 nM RAPA significantly reduced HMC3 protein content, both in resting cells as well as in cells exposed to II. Data are means ± SEM (n=15) and were analyzed by one-way ANOVA followed by the Bonferroni's *post hoc* test. ***, *P*<0.001, versus Control. (B) The effect of different treatments on cell viability was assessed by the MTS reduction assay. II did not change HMC3 cell viability nor did treatment with RAPA. Results are shown as fold changes versus Control, set as 100 %. Results are pooled analysis of three independent experiments (n= 4 per experimental group). Data are means ± SEM (n=12) and were analyzed by one-way ANOVA followed by the Bonferroni's *post hoc* test. (C) The cytotoxic effect of the treatments was assessed by measuring the release of LDH in the incubation medium, taken as an index of cell toxicity. None of the treatments was toxic for HMC3 microglial cells. Results are shown as fold changes versus Control, whose optical density was set as 100 %. Results are pooled analysis of three independent experiments (n= 3-6 per experimental group). Data are means ± SEM (n=12) were analyzed by one-way ANOVA followed by the Bonferroni's *post hoc* test.

The inhibitory effect of RAPA on ROS production was paralleled by a significant reduction of the total protein content (Figure 5A(Fig. 5)). Similar reductions were observed in the experiments in which IL-6 release was assessed (data not shown). Therefore, reductions in the intracellular protein content would reduce/abate the inhibitory action of RAPA on ROS production if protein levels were used to normalize the data. On the other hand, the stimulatory effects of RAPA on IL-6 production would be significantly increased by normalization using total intracellular protein content. Interestingly, Dex, within the concentration range of 0.1-10 µM, did not modify total protein content (Supplementary Figure 1D). Reductions in the intracellular protein levels may be consistent with the well characterized inhibitory activity of RAPA on protein translation (Laplante and Sabatini, 2012[24]). However, in order to exclude that the effects of RAPA on ROS production and protein synthesis were merely due to cytotoxicity, we evaluated cell viability after treatments with two different assays (Dello Russo et al., 2009[12]). Consistently with our previous observations (Dello Russo et al., 2009[12]), II tended to increase human microglial viability albeit to a lower extent and the effect was not significant. Moreover, RAPA (in the range of concentrations 0.1-10 nM) tended to reduce microglial viability back to control levels in cells activated with II. These observations are in line with our previous data obtained using rat primary microglial cultures exposed to a mixture of pro-inflammatory cytokines that included also TNFα (Dello Russo et al., 2009[12]). In contrast to rodent cells, RAPA did not significantly affect human microglial cell viability under basal conditions (Figure 5B(Fig. 5)). Taken together these data suggest that human microglial cells, in part due to the immortalization procedure, are more resistant to the metabolic/cytotoxic effects of RAPA. This was further confirmed by using a different cytotoxicity assay. As shown in Figure 5C(Fig. 5), RAPA did not significantly increase the release of lactate dehydrogenase by HMC3 microglial cells neither under basal conditions nor in cells activated with II.

### Rapamycin effects on cell morphology and proliferation

**Figure 6:**
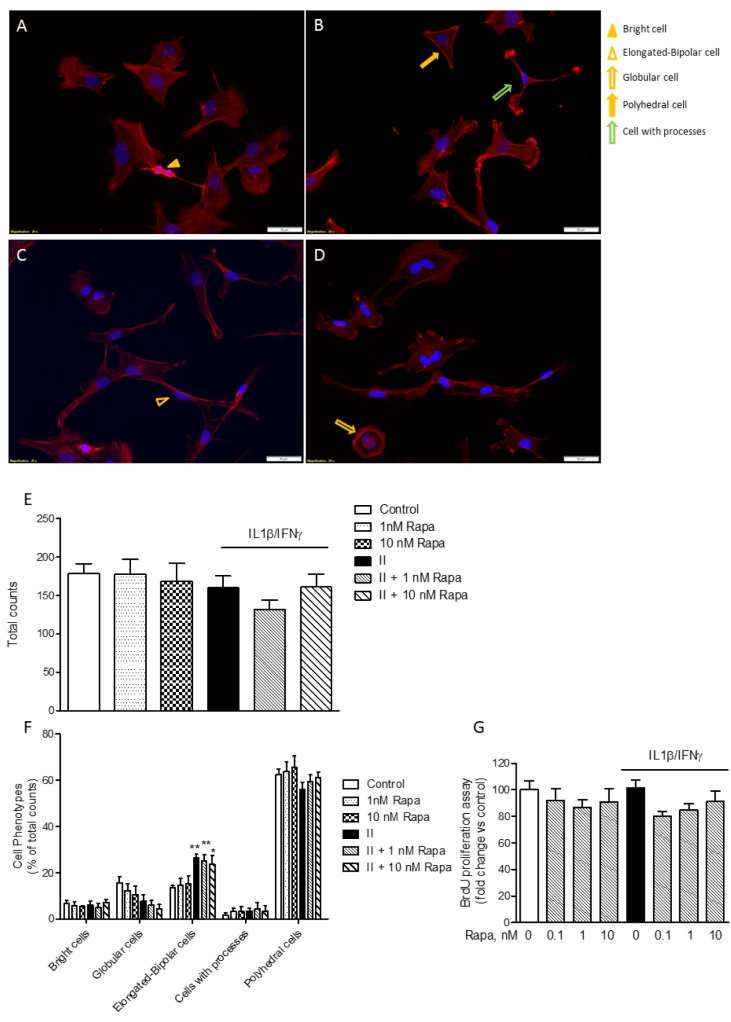
Rapamycin effect on HMC3 cell morphology and proliferation. (A-D) HMC3 cells were grown on glass coverslips, let recover overnight and treated for 24 h with plain medium as Control (A); 1 nM RAPA (B); II (C); II in combination with 1 nM RAPA (D). Cell morphology was evaluated by labeling the cytoskeletal F-actin filaments with tetramethylrhodamine (TRITC)-conjugated phalloidin (red fluorescence). Cells were counterstained with the nuclear probe, 4′,6-diamidino-2-phenylindole dihydrochloride (DAPI, blue fluorescence). The morphology of HMC3 cells is shown on representative merged images. The experiment was carried out in duplicate and repeated three times with similar results. Different cell shapes were identified as indicated in the images and reported in the legend on the right: bright cells (yellow arrow-head) (A); elongated-bipolar cells (empty yellow arrow-heads) (C); globular-shaped cells (empty yellow arrow) (D); polyhedral-shaped cells (yellow arrows) (B); cells extending processes (empty green arrow) (B). The morphology of HMC3 appeared to be complex, including cells with different shapes. However, cells with polyhedral and globular shapes prevailed in Control, whereas an increased number of elongated-bipolar cells was found after treatment with II. Scale bar 50 μm (A-D). (E) Cells were manually counted in 15 different fields by two independent investigators and average cell count is shown in the graph. No significant differences in the total number of cells were observed due to different treatments. (F) Cells with different shapes were counted in 15 different fields. The data were normalized by total cell count per each treatment (E) and results are shown in the graphs. II significantly increased the percentage of elongated-bipolar cells, whereas it reduced the proportion of cells with polyhedral and globular shapes. RAPA per se slightly reduced the percentage of globular cells, whereas it did not significantly modify the effect of II. (E-F) Results are from three independent experiments performed in duplicate. Data are means ± SEM and were analyzed by one- or two-way ANOVA followed by the Bonferroni's *post hoc* test. *, *P<*0.05; **, *P*<0.01, versus Control. (G) HMC3 cells were stimulated with II for 24 h. RAPA in the concentration range 0.1-10 nM was added at the beginning of the experiments. The effect of different treatments on cell proliferation was assessed by 5-bromo-2-deoxyuridine (BrdU) incorporation. Results are shown as percentage of BrdU incorporation in the Control (set as 100 %). Pooled analysis of two independent experiments (n= 4-5 per experimental group) is reported. Data are means ± SEM (n=9) and were analyzed by one-way ANOVA followed by the Bonferroni's *post hoc* test.

As shown in Figure 6A-D(Fig. 6), the HMC3 cells displayed a complex morphology. Under basal conditions, globular cells (*i.e.*, flat cells with discrete cytoplasm) (Dello Russo et al., 2018[11]) can be more precisely distinguished in cells with a polyhedral shape (*i.e.* straight margins) and cells with a more round/globular cytoplasm (Figure 6A(Fig. 6)). The most prevalent phenotype under basal conditions included polyhedral cells (Figure 6F(Fig. 6)). On the other hand, in response to II there was a significant increase in the number of elongated cells (as shown in Figure 6C(Fig. 6) and quantitated in Figure 6F(Fig. 6)). In addition, a few numbers of very bright cells with scarce cytoplasm (Figure 6A(Fig. 6)) and cells with a more branched morphology (*i.e.* cells with processes, Figure 6B(Fig. 6)) were also detected in this more detailed analysis of cell phenotypes (Dello Russo et al., 2018[11]). Interestingly, RAPA did not exert any significant effect on cell morphology neither under basal conditions nor after stimulation with II (Figure 6B, D and E(Fig. 6)). Inhibition of mTOR kinase activity is often associated with reduced cell proliferation (Tian et al., 2019[46]). However, we did not detect any significant reduction in the total number of cells in response to RAPA used alone or in combination with II (Figure 6E(Fig. 6)). Consistently, we did not observe any significant inhibitory effect on microglial cell proliferation after 24 h incubations (Figure 6G(Fig. 6)). However, when cells were incubated for longer times (48 h), II tended to reduce cell proliferation but this effect was not further increased by exposure to RAPA (data not shown). This observation is in contrast with the anti-proliferative effects of RAPA observed in primary cultures of rat microglial cells (Dello Russo et al., 2009[12]). 

## Discussion

In the present paper we have shown that the human microglial HMC3 cell line produces sizable amount of IL-6 and ROS under basal conditions. The production of IL-6 significantly increased in response to pro-inflammatory cytokines, being the combination of IFNγ and IL-1β to most effective stimulus. This mixture also significantly increased the mRNA expression level of IL-6. Both IL-6 and ACTB Q-PCR amplification products were sequenced and proved to align to the proper genomic DNA reference sequence, which provides validation of the primer sets used for the Q-PCR analysis. On the other hand, pro-inflammatory cytokines (alone or in combination with IFNγ) did not affect ROS production in the HMC3 cells. Interestingly, II increased the percentage of elongated cells within the culture, suggesting that this phenotype can possibly relate to the activation state of microglia. When tested in this experimental model, RAPA significantly increased the stimulatory effect of II on IL-6, thus promoting a further increase in the expression level of this cytokine as well as in the amount secreted in the incubation media. However, RAPA reduced ROS production both under basal conditions and in cells activated with II. Consistently with its known mechanisms of action, RAPA reduced the extent of mTORC1 activation and the total amount of intracellular proteins in microglial cells. However, the drug did not alter microglial cell viability nor inhibited cell proliferation. Finally, RAPA did not display any significant effect on cell morphology. 

As reported in the introduction, there is conflicting evidence from rodent studies about the pharmacological effects of RAPA on the regulation of microglial inflammatory responses. Taken together, data presented in this paper confirm the complexity of the pharmacological effects of RAPA in human microglia as well. The drug significantly increased the production of IL-6 in HMC3 microglial cells activated with II. Albeit other inflammatory genes were not modulated by RAPA, these data would suggest that inhibition of mTORC1 in human microglia holds the potential to increase their pro-inflammatory activation. Consistently, it has been shown that inhibition of mTOR increases IL-6 production in isolated human peripheral blood monocytes activated with different bacterial derived activators (Weichhart et al., 2008[48]). However, the pro-inflammatory activity of RAPA in these cells was broader, including increased production of other pro-inflammatory cytokines (namely IL-12, IL-23 and TNFα) and reduced release of the anti-inflammatory cytokine IL-10. Increased secretion of pro-inflammatory cytokines was mediated by activation of NFkB (nuclear factor-kB) whereas inhibition of IL-10 resulted by inhibition of STAT3 (signal transducer and activator of transcription 3) (Weichhart et al., 2008[48]). Similarly, RAPA increased TNFα production in peritoneal activated macrophages, via inhibition of IL-10 production (Baker et al., 2009[3]). RAPA also reduced the production of IL-10 by activated primary human monocytes, particularly in CD14 highly positive cells (Lenart et al., 2017[26]). Pro-inflammatory properties of RAPA were also observed in peripheral blood monocytes stimulated with LPS, in which the drug increased the release of IL-12 and reduced the secretion of IL-10 (Schmitz et al., 2008[43]). However, in this model other pro-inflammatory markers (such as TNFα, IL-6, and NO) were reduced by RAPA treatment (Schmitz et al., 2008[43]). Interestingly, pretreatment of LPS-stimulated monocytes with RAPA increased their ability to stimulate the secretion of pro-inflammatory cytokines, such as IFNγ and IL-17, by T cells; whereas RAPA directly inhibits T cell proliferation and IFNγ release (Weichhart et al., 2008[48]). These data would therefore suggest that mTOR is involved in the regulation of macrophage function, by favoring the acquisition of an alternative (M2) status of activation. In support of this hypothesis, it has been shown that the bone morphogenetic protein-7 (BMP-7) mediates monocyte polarization into M2 macrophages through the activation of the PI3K-AKT-mTOR pathway (Rocher and Singla, 2013[40]); and RAPA, by blocking mTOR, unbalances the polarization of human macrophages towards the classic (M1) status of activation (Mercalli et al., 2013[33]). Moreover, pro-inflammatory effects were also observed using RAPA in dendritic cells (DCs), another cardinal phagocyte cell type of the innate immune system, whereas stimulation of toll like receptors (TLRs) by different PAMPs (pathogen associated molecular patterns) increases both mTORC1 and 2 activity via activation of a PI3K mediated signaling pathway (Weichhart et al., 2008[49]; Schmitz et al., 2008[43]). In addition, RAPA inhibited the complete development of DCs, both in mouse and human cells (Katholnig et al., 2013[22]). However, in this paper, anti-inflammatory effects of RAPA were observed in macrophages. Furthermore, constitutive activation of mTORC1 has been shown to impair IL-4-induced M2 macrophage polarization and increase inflammatory responses to pro-inflammatory stimuli (Byles et al., 2013[6]). On the other hand, RAPA has been shown to reduce the expression of M2 polarization activation markers (including ARG-1, CD163 and CD206) in macrophages (Lim et al., 2017[28]). 

However, other studies showed that mTOR inhibition decreases the global inflammatory response of *in vitro* generated DCs (Monti et al., 2003[34]; Fischer et al., 2009[13]) and reduces the production of nitric oxide (NO) by macrophages activated with the bacterial endotoxin lipopolysaccharide (LPS) (Weinstein et al., 2000[50]). In peripheral blood monocytes, RAPA increased the release of IL-12 and reduced production of IL-10 in response to, but increased the expression of other pro-inflammatory markers (such as TNFα, IL-6 and nitric oxide, NO) (Schmitz et al., 2008[43]). Inhibitory effects of RAPA on basal IL-6 production and other inflammatory genes in basal conditions (IL-1β, IL-8 and TNFα) were observed in the human monocyte cell line THP-1 as well as THP-1 derived macrophages (Xie et al., 2014[51]). In these cell lines, anti-inflammatory effects of RAPA were also observed in cells activated with polystyrene nanoparticles with carboxyl groups (Loos et al., 2014[31]) and in cells exposed to bacterial peptidoglycan (Lee et al., 2015[25]). Anti-inflammatory actions of RAPA were also observed in macrophages co-infected with HIV and Mycobacterium tuberculosis (Andersson et al., 2016[1]), as well as in monocyte activated with LPS and other metabolites of cholesterol (Bekkering et al., 2018[4]). Interestingly, in the human microglial cell line (HM-SV40) and in primary cultures of human microglia, inhibitory effects of RAPA were reported on the expression of several cytokines and chemokines, *i.e.* IL1β, IL8, CCL2 and CCL5, in response to neurotensin (Patel et al., 2016[37]). In this experimental paradigm, cells were pre-treated with 100 nM RAPA for 2 h and then stimulated with 10 nM neurotensin for 12 or 24 hours in serum-free medium. The authors observed increased expression (at 12 h) and release (at 24 h) of the above mentioned pro-inflammatory cytokines. Notably, IL-6 was not evaluated in this set of experiments. Moreover, neurotensin significantly increased the rate of microglial proliferation after 48 h and RAPA (at 500 nM) displayed inhibitory effects. However, it has been shown that at higher concentrations and for prolonged treatment RAPA may interfere with the activation of mTORC2 as well (Sarbassov et al., 2006[42]), and this may contribute to the inhibitory effects of RAPA observed in this experimental model. Consistently, similar inhibitory actions were observed in this experimental model using an mTORC1-mTORC2 dual inhibitor. Taken together, these data suggest that the role of mTOR and the resulting effects of its inhibition in the regulation of innate immune cell responses are complex. In fact, the final outcome of RAPA treatment may depend on the inflammatory environment as well as on the inflammatory stimuli.

Notably, when used *in vivo* as immunosuppressant in patients undergoing organ transplantation, RAPA induces a state of increased inflammatory activation of the innate immune system. Increased pro-inflammatory activation induced by RAPA were only in part reversed by glucocorticoids, like Dex. Moreover, while RAPA and Dex displayed synergistic immunosuppressive effects on human T cells, RAPA antagonized the antinflammatory effects of Dex on human peripheral blood monocytes (Weichhart et al., 2011[49]). When tested in our experimental model, Dex significantly reduces the production of IL-6 in microglial cells activated with II. Moreover, while RAPA reduced the extent of microglial ROS production, both under basal conditions and in cells activated with II, Dex did not modify ROS production. In this regard, it has been shown that constitutive ROS production is an important intracellular mechanism that controls cell proliferation (Pani et al., 2000[35]). In line with this evidence, we found that the production of ROS in microglial cells was inversely correlated to the initial density of plating (data not shown). However, despite a significant reduction of protein intracellular content in response to RAPA, we did not observe any significant toxic effect of RAPA on HMC3 cell viability. Consistently, RAPA did not interfere with HMC3 cell proliferation nor with its morphological properties. In this study, we also provided a more detailed characterization of HMC3 microglial morphology. Under basal conditions, we observed mostly flat cells with polyhedral or round shapes and quite large cytoplasm. Few cells had reduced cytoplasm and stained highly positive with rhodamine-phalloidin. On the other hand, activation of HMC3 cells with II resulted in a significant increase of cells with elongated shapes, which may suggest a different status of activation. RAPA did not significantly modify microglial morphology.

In conclusion, data presented in this paper suggest that RAPA modulates the activation of microglial cells, increasing their pro-inflammatory activity. This finding may be particularly relevant in consideration of the recent increasing interest in testing RAPA in a variety of human neurological conditions (Crino, 2016[8]).

## Acknowledgement

The present research is funded by Angelini S.p.A. and by Fondi di Ateneo 2017, Università Cattolica del S. Cuore, both grants awarded to CDR. Data were timely shared with Angelini S.p.A. through progress and final reports, and during one internal meeting.

## Conflict of interest

The present research was in part funded by Angelini S.p.A., grant awarded to Dr Cinzia Dello Russo. Dr Isabella Coletta is employee of Angelini S.p.A. and provided scientific advice on the project. All the other authors declare that they have no conflicts of interest that might be relevant to the contents of this manuscript.

## Supplementary Material

Supplementary material
