# Supplementary material for: The mTOR kinase inhibitor rapamycin enhances the expression and release of pro-inflammatory cytokine interleukin 6 modulating the activation of human microglial cells
Source: EXCLI J. 2019 Sep 6;18:779–98. doi: 10.17179/excli2019-1715 (PMC6806201; doi:10.17179/excli2019-1715)
Supplement: Supplementary material [file EXCLI-18-779-s-001.pdf]

EXCLI Journal 2019;18: 779-798 – ISSN 1611-2156
Received: August 07, 2019, accepted: September 02, 2019, published: September 06, 2019

Supplementary material to:

THE mTOR KINASE INHIBITOR RAPAMYCIN ENHANCES THE EXPRESSION AND RELEASE OF PRO-INFLAMMATORY CYTOKINE INTERLEUKIN 6 MODULATING THE ACTIVATION OF HUMAN MICROGLIAL CELLS

Natalia Cappoli[1], Daniele Mezzogori[2], Elisabetta Tabolacci[3], Isabella Coletta[4], Pierluigi Navarra[5], Giovambattista Pani[6], Cinzia Dello Russo[5*]

[1] Institute of Pharmacology, Università Cattolica del S. Cuore, Roma
[2] Institute of Human Physiology, Università Cattolica del S. Cuore, Roma
[3] Fondazione Policlinico Universitario A. Gemelli IRCCS, Roma -Institute of Genomic Medicine, Università Cattolica del S. Cuore, Roma
[4] Angelini RR&D (Research, Regulatory & Development) - Angelini S.p.A.
[5] Pharmacology Unit, Fondazione Policlinico Universitario A. Gemelli IRCCS, Roma – Institute of Pharmacology, Università Cattolica del S. Cuore, Roma
[6] Fondazione Policlinico Universitario A. Gemelli IRCCS, Roma – Institute of General Pathology, Università Cattolica del S. Cuore, Roma

* Corresponding author: Cinzia Dello Russo; Institute of Pharmacology, Università Cattolica del S. Cuore, L.go F Vito 1, 00168 Rome; Tel. 0039-06-30154367; Fax: 0039-06-3050159; E-mail: cinzia.dellorusso@unicatt.it

http://dx.doi.org/10.17179/excli2019-1715

This is an Open Access article distributed under the terms of the Creative Commons Attribution License (http://creativecommons.org/licenses/by/4.0/).

EXCLI Journal 2019;18: 779-798 – ISSN 1611-2156
Received: August 07, 2019, accepted: September 02, 2019, published: September 06, 2019

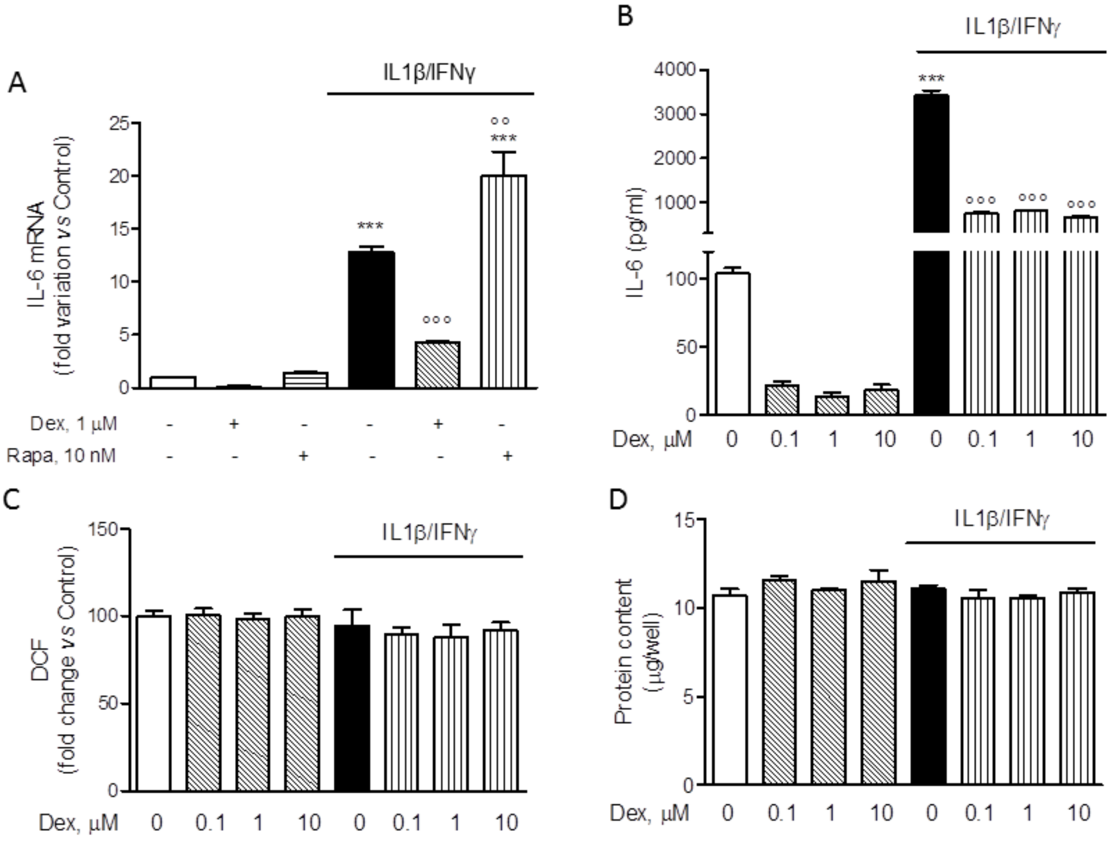

Supplementary Figure 1: Effect of dexametasone on HMC3 cell inflammatory activation and protein synthesis. (**A-D**) The effects of a well-known anti-inflammatory drug, dexamethasone (Dex) were evaluated on HMC3 proinflammatory activation. (**A**) Cells were treated in plain medium (Control) or stimulated with II for 4 h. As indicated, 1 µM Dex and 10 nM RAPA (for comparison) were added at the beginning of the experiment. Total cytosolic RNA was prepared and used for Q-PCR analysis of IL-6 gene. Data are expressed as fold change versus Control, taken as calibrator for comparative quantitation analysis of mRNA levels. As shown in the graph, II significantly increased IL-6 mRNA levels as early as after 4 h of incubation. Dex completely abolished IL-6 expression under basal conditions and significantly reduced the induction obtained with II. On the other hand, RAPA significantly increased the stimulatory effect of II (consistently with results reported at 24 h). Each sample was measured in triplicate, the experiment was repeated two times with similar results. Data are means ± SEM and were analyzed by one-way ANOVA followed by the Bonferroni's *post hoc* test. ***, $P<0.001$, versus Control; °°°, $P<0.001$, °°, $P<0.01$, versus II. (**B-C**) Microglial cells were treated in plain medium (as Control) or stimulated with II for 24 h. Dex in the concentration range of 0.1-10 µM was added at the beginning of the experiment. (**B**) The amount of IL-6 released in the incubation media by HMC3 cells, under the different experimental conditions, was measured. Consistently with mRNA data, II significantly increased the release of IL-6 in the incubation media. In contrast to RAPA, Dex significantly reduced the stimulatory effect of II (at any level of concentration tested). Data are shown as pg/ml. Data are means ± SEM (n= 4). The experiment was repeated three times using cells from two different thaws with similar results. Data were analyzed by one-way ANOVA followed by the Bonferroni's *post hoc* test. ***, $P<0.001$, versus Control; °°°, $P<0.001$, versus II. (**C**) Reactive free radicals were assessed by the oxidation of H2DCF-DA (DCF) added to the cells at the end of the experiment. Data are expressed as fold change versus Control (set as 100 %). Data are means ± SEM (n= 4). The experiment was repeated three times using cells from two different thaws with similar results. Data were analyzed by one-way ANOVA followed by the Bonferroni's *post hoc* test. (**D**) At the end of experiments in which ROS production was assessed (**C**), cells were lysed in 200 mM NaOH and protein content was evaluated by the Bradford's method. Data are means ± SEM (n= 4). The experiment was repeated three times using cells from two different thaws with similar results. Data were analyzed by one-way ANOVA followed by the Bonferroni's *post hoc* test.
